# Iron-Modified Acid Carbons for the Conversion of Fructose to 5-Hydroxymethylfurfural under Microwave Heating

**DOI:** 10.1021/acsomega.4c07030

**Published:** 2024-11-01

**Authors:** Letícia F. L. Machado, Luana S. Andrade, Dalmo Mandelli, Wagner A. Carvalho

**Affiliations:** † Center for Natural Sciences and Humanities, Federal University of ABC (UFABC), Av. dos Estados, 5001, Santo André, SP CEP 09210-580, Brazil; ‡ Department of Chemistry, Northwestern University, 2145 Sheridan Road, Evanston, Illinois 60208, United States

## Abstract

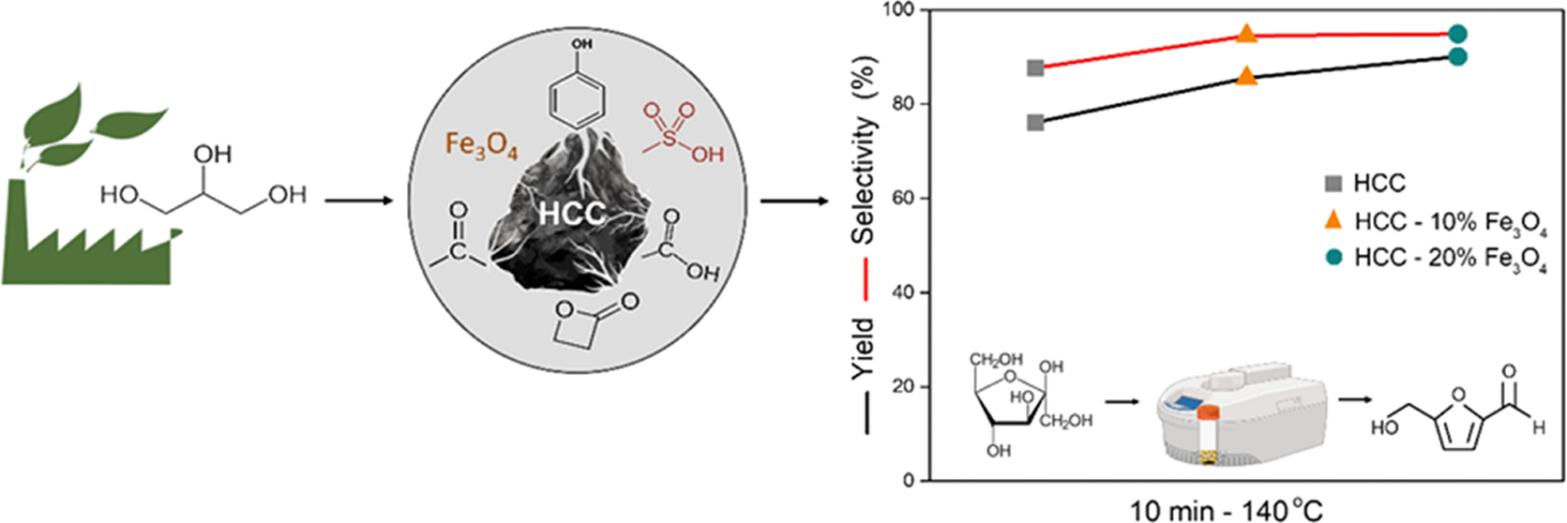

Carbons with Brønsted acidic sites and iron oxide modifications were prepared through hydrothermal carbonization and glycerol pyrolysis in the presence of sulfuric acid, magnetite, and iron(III) nitrate. The solids were tested as catalysts in converting fructose to 5-hydroxymethylfurfural (5-HMF). Characterization techniques revealed a uniform presence of 4.89 mmol g^–1^ total acidic groups, including up to 1.87 mmol g^–1^ sulfonic and carboxylic groups. Combined with a reduced surface area, the Brønsted and Lewis acidity enabled the conversion of 94% of fructose with selectivity values as high as 95% for 5-HMF in just 10 min at 140 °C, using microwave heating and dimethyl sulfoxide (DMSO) as the solvent. This performance was attributed to the selective heating of the catalyst surface by the microwave absorption capacity of the acidic groups and iron oxide, leading to the formation of “hot spots.” The catalyst obtained by hydrothermal carbonization in the presence of Fe_3_O_4_, HCC-20% Fe_3_O_4_, demonstrated stability when reused for up to four consecutive cycles. A slight reduction in conversion and selectivity was observed after the first use, attributed to the presence of acid species not incorporated into the solid during the synthesis process.

## Introduction

The growing demand for materials and energy from fossil sources raises concerns about sustainability and environmental impact.^1^ Biomass, a renewable resource, has emerged as a promising alternative. The valorization of biomass to produce high-value products, such as 5-hydroxymethylfurfural (5-HMF), obtained from the dehydration of fructose, has shown promising results.^2^ 5-HMF is recognized as a key chemical platform with considerable industrial and economic significance, because it can be chemically modified to form essential building blocks for various industries, including polymers and biofuels.^3^ Traditionally, inorganic acids like HCl, HNO_3_, H_3_PO_4_, and H_2_SO_4_ are used as catalysts, but their application is hazardous and costly. In contrast, chemically modified carbons with acidic surface groups can serve as viable and safer substitutes.^4,5^ These carbons can be produced using different methodologies, notably hydrothermal carbonization (HTC) and pyrolysis.^6,7^ The HTC method is more cost-effective and efficient since it requires less time and temperature, allows wet biomass use, and minimizes waste production.^8^ On the other hand, pyrolysis offers greater surface area development, control over pore structure through chemical activation, and solids with higher fixed carbon content due to the high temperature used.^9^ Both methods have advantages and disadvantages depending on the specific process needs.

The use of glycerol as a carbonaceous precursor offers significant economic and environmental advantages. During the transesterification process for biodiesel production, glycerol is generated as a coproduct in a weight ratio of approximately 1:9 relative to biodiesel.^10^ The rapid growth of the biodiesel industry has led to a substantial surplus of glycerol, with supply far exceeding demand in its traditional markets, such as the food, cosmetics, and pharmaceutical industries. Since 1995, the market has consistently faced an oversupply of glycerol, with current estimates indicating that supply is roughly six times higher than demand. This surplus highlights the need for alternative applications to increase the value of this abundant coproduct.^11^ Utilizing excess glycerol for carbon production not only recycles industrial waste but also helps reduce pollution, contributing to sustainability efforts.^12^ The low cost of glycerol combined with the presence of three hydroxyl groups in its structure makes it an attractive material for dehydration and polymerization. When in contact with acid, especially H_2_SO_4_, the hydroxyl groups become protonated, which facilitates the dehydration reactions and promotes the formation of structures like polyethers. These structures not only increase the hydrophilicity of the solid but also enable the incorporation of acidic functional groups such as sulfonic, carboxylic, and phenolic when exposed to moderate heating.^13,14^

The presence of these acidic sites on the catalyst’s surface is essential for promoting the three dehydration steps of fructose required to produce 5-HMF.^15^ Although sulfonic groups (–SO_3_H) are frequently highlighted in the literature,^6^ carboxylic (–COOH) and phenolic (–OH) groups^16,17^ can also act synergistically with the solvent dimethyl sulfoxide (DMSO), forming the catalytic species [DMSOH]^+^, which plays a key role in enhancing the efficiency of the dehydration process.^18^

Dimethyl sulfoxide (DMSO) is one of the most widely used solvents due to its high substrate solubility, which favors its more reactive form for the desired conversion, fructofuranose, as well as stabilizing 5-HMF and minimizing unwanted secondary reactions.^19^ Additionally, DMSO is valued for its low toxicity and cost, and there is evidence that it may exhibit catalytic performance beyond its role as a solvent.^20,21^ Another obvious solvent choice is water, which not only solubilizes the substrate but can also generate hydronium ions that catalyze the reaction. However, yield and selectivity for the platform product are lower with water compared to DMSO. In aqueous media, 5-HMF tends to undergo rehydration, leading to the formation of byproducts like formic acid and levulinic acid.^22^

The heating method plays a significant role in the catalytic outcome, with microwave heating offering distinct advantages over conventional methods despite being less explored. The main advantage of microwave heating lies in its direct interaction with reactant components through a dielectric process. Microwaves (MW) can penetrate materials, delivering energy that is converted into heat throughout the entire volume of the material, a process known as volumetric heating.^23^ This contrasts with conventional heating, where higher temperatures occur at the container walls and outer surfaces due to conduction and convection.^24,25^ Some catalysts can effectively absorb microwaves and generate temperatures higher than the surrounding environment, creating “hot spots.” These hot spots result from molecular-level disruptions in the internal composition of the cells, leading to an increased mass transfer rate. Kong et al.^26^ and Ji et al.^27^ demonstrated the formation of these hot spots through surface polarization by incorporating components with dielectric properties, such as sulfonic groups. The acidic site’s presence increases microwave absorption and the heat transfer efficiency on the catalyst’s surface. As a result, there is a significant improvement in the energy efficiency of the fructose dehydration reaction due to increased molecular contact. The rise in surface heat amplifies molecular vibrations, enhancing collisions between the reactant and product molecules. To validate the statement, Kong et al. performed fructose dehydration studies at the same temperature and time: 186 °C for 10 min. Conventional heating yielded 47.2% of 5-HMF. In contrast, microwave heating produced a yield of 88.3%.^26^ Ji et al. found that adding Nb to TiO_2_ solid acid catalysts improved the material’s acidity. This action boosted electron transfer from the catalyst to the sulfonic groups, which increased the compound’s acidity and polarity. The increase in polarity increased heat generation on the catalyst’s surface, resulting in a 32% higher reaction rate.^27^ Lyu et al.^28^ highlighted the efficiency of carboxylic and sulfonic groups in response to microwave absorption. When synthesizing acid char derived from glucose, they observed that achieving a yield of 86% of 5-HMF under conventional heating at 70 °C required 300 min. In contrast, under the same temperature conditions with microwave heating, they obtained a yield of 89.2% in just 5 min.^28^

Considering the benefits provided by polarization, we decided to evaluate the combined use of iron with acidic sites to enhance the catalytic performance. The introduction of iron can increase the dielectric properties of carbon surfaces as studies indicate that iron oxide exhibits multiple characteristics of reflection and polarization. When exposed to electromagnetic fields, such as microwaves, the particles respond with various energy dissipation mechanisms including polarization and multiple relaxations. These processes optimize microwave absorption, leading to a more efficient conversion of electromagnetic energy.^29,30^

This study aims to demonstrate that iron oxide and Brønsted acid sites absorb radiation, creating hot spots that raise surface temperatures. This increase in the surface temperature boosts the kinetic energy of substrate molecules, enhancing their interaction with the Brønsted catalytic sites on the catalyst. The combination of microwaves, Brønsted acid sites, and iron oxide improves fructose dehydration, leading to the production of 5-hydroxymethylfurfural.

## Experimental Section

### Reagents

Glycerol (Synth, 99.5%), sulfuric acid (Synth, 95–98%), iron(III) nitrate nonahydrate (Synth, 98%), nitric acid (Synth, 65%) commercial magnetite (SkySpring Nanomaterials, 20–30 nm, 98%), acetone (Synth, 99.5%), sodium hydroxide (Synth, 98%), sodium bicarbonate (Sigma-Aldrich, ≥99,7%), hydrochloric acid (37% P.A.), d-(−)-Fructose (Sigma-Aldrich, ≥99%), and dimethyl sulfoxide (Synth, ≥99.9%) were used as received, without further purification.

### Catalyst Preparation

For the hydrothermal carbonization process, 5 g of glycerol was homogenized with commercial magnetite in mass ratios of 10 and 20% using a mechanical stirrer at 100 rpm for 10 min. After adding 30 g of H_2_SO_4_, the mixtures were thermally treated at 180 °C for 15 min.^4,31^ These solids were identified as HCC-10% Fe_3_O_4_ and HCC-20% Fe_3_O_4_, respectively. For comparison, a solid without magnetite was synthesized and identified as hydrothermal carbonization carbon (HCC).

In the pyrolysis process, 10 g of glycerol were homogenized under the same conditions as mentioned earlier with an aqueous solution of Fe(NO_3_)_3_·9H_2_O 0.36 mol L^–1^, in an amount equivalent to 5 wt % of iron relative to glycerol. The mixture was heated in a tubular furnace under N_2_ atmosphere at a heating rate of 30 °C min^–1^, until 380 °C for 180 min.^32^ The resulting solid, termed CP (Pyrolysis Carbon), was ground, and 1 g of the material was mixed with 10 mL of H_2_SO_4_ and heated in an autoclave for 2 h at 180 °C under N_2_ atmosphere and autogenous pressure.^33^ This carbon was identified as CPS (Sulfonated Pyrolysis Carbon). Solids were washed with deionized water and acetone using a Soxhlet system.

### Catalyst Characterization

Surface acidity was quantified according to the Boehm method.^34,35^ For the test, 300 mg of carbon was added to 25 mL of 0.1 mol/L NaOH to quantify all Brønsted acid groups on the carbon surface or to 25 mL of 0.1 mol/L NaHCO_3_ to quantify sulfonic and carboxylic acid groups. A blank was prepared for each experimental condition without adding carbon. The suspensions were degassed with N_2_, sealed in bottles, and shaken for 24 h at room temperature. Aliquots of the filtered solutions were titrated with 0.1 mol/L HCl by using a Metrohm 905 Titrando automatic titrator. Infrared analyses were conducted with a Varian-Agilent 640-IR FTIR spectrometer in the attenuated total reflectance (ATR) mode. Elemental analyses of the carbons were performed by using a Thermo Scientific Flash EA 1112 Elemental Analyzer. Micrographs were obtained in a compact JEOL JSM-6010LA scanning electron microscope operating at 15 kV. X-ray diffraction patterns were acquired using a Bruker AXS D8 Focus diffractometer in the 2θ range of 5–80° with a step size of 0.02 per second. Nitrogen adsorption and desorption isotherms were obtained by using a Quantachrome Autosorb 1-MP surface area analyzer.

Iron quantification in the carbon matrix was performed by inductively coupled plasma optical emission spectrometry (ICP-OES) using a Thermo iCap7600 ICP-OES system. In this analysis, approximately 3 mg of the sample were digested with 2 mL of concentrated nitric acid under microwave heating (Biotage Initiator) for 15 min at 180 °C. X-ray photoelectron spectroscopy (XPS) measurements were conducted in a Thermo Scientific ESCALAB 250 Xii (Al Kα radiation, *h*ν = 1486.6 eV). XPS data were analyzed using the Thermo Scientific Avantage Software, with spectra calibrated referencing the C 1s peak at 284.8 eV. Thermogravimetric analyses (TG/DTG) were performed using a TA Instruments Discovery TGA. The solids were evaluated under N_2_ from 25 to 800 °C with a heating rate of 10 °C min^–1^.

### Fructose Dehydration

**Figure 1:**
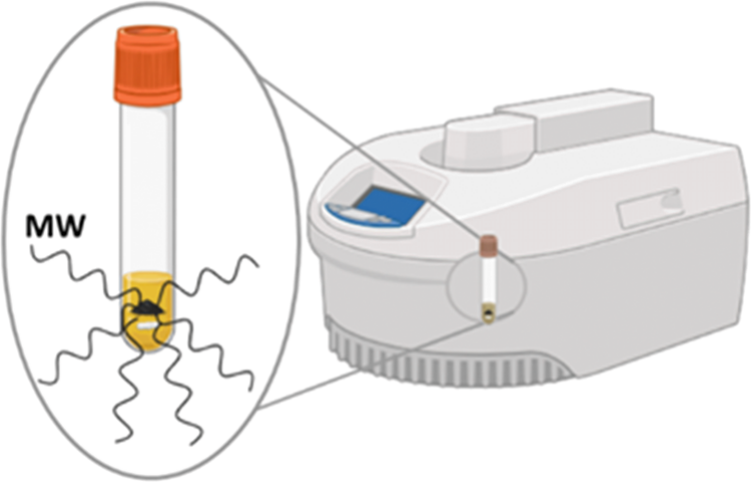
Illustrative figure of the microwave (MW) reactor used in the dehydration of fructose.

The catalytic tests were conducted using the CEM Discover 2.0 microwave reactor, a monomode system operating at 2.45 GHz, ensuring a uniform energy distribution within the cavity. Heating occurs volumetrically and instantaneously. The reactor is equipped with an infrared sensor that directly measures the sample’s temperature through the glass and Teflon, materials used to contain the reaction mixture and ensure system safety. Figure 1 illustrates the reactor, highlighting the volumetric heating of the reaction mixture.

Initial heating occurred with a power ranging from 0 to 200 W for 2 min until the desired temperature was reached. This temperature was then maintained by applying a power range of 20–40 W. Reactions were conducted with 5 mL of a 5 wt % fructose in DMSO and 5 wt % of catalyst relative to fructose, in a glass reactor containing a Teflon-coated magnetic stir bar. Reaction times varied from 3 to 30 min. Blank tests were carried out in the absence of the catalyst. Product identification and quantification were performed by high-performance liquid chromatography (HPLC) using an Agilent 1220 Infinity LC equipped with a Rezex ROA Organic Acid H+ (8%) column (250 mm × 4.6 mm) maintained at 60 °C. Reaction products were identified and quantified using pure standards and analytical curves for fructose, 5-HMF, levulinic acid, and formic acid. Fructose was quantified using a refractive index detector (RID) at 40 °C, while 5-HMF and formic and levulinic acids were detected using a diode array detector (DAD) at 254 and 210 nm, respectively. The fructose conversion and the yield and selectivity of the obtained products were calculated using eqs 1–3, respectively. Additionally, the turnover number (TON) and turnover frequency (TOF) were determined using eqs 4 and 5. The mass of 5-HMF produced was calculated by multiplying its molar mass (126.11 g/mol) by the number of moles identified via HPLC.
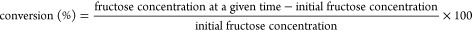
1
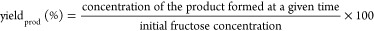
2
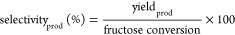
3
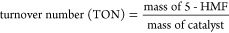
4
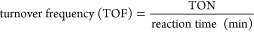
5

### Catalyst Recycling

Recycling tests were conducted under the conditions previously optimized in this study. After each reaction cycle, the catalyst was recovered by centrifugation, washed with water and acetone, and dried at 100 °C for 24 h before the next application.

### Leaching Test

In a procedure similar to the catalytic activity test but without reaching equilibrium and without adding the substrate, the solvent and catalyst were heated for 3 min at 140 °C. The catalyst was removed from the reaction medium using a syringe equipped with a 0.45 μm filter. The substrate was then added to the liquid phase and briefly homogenized using a vortex before being subjected to the same reaction conditions. The procedure was repeated by using the same catalyst to evaluate the potential for leaching in subsequent reactions.

## Results and Discussion

### Characterization

The synthesis yield varied significantly, depending on the method used. Hydrothermal carbonization produced solids with mass yields ranging from 48 to 52%. In contrast, pyrolysis resulted in considerably lower yields, approximately 8% after the initial thermal treatment and 3% after sulfonation. Similar results for hydrothermal carbonization of glycerol have been reported in the literature, reinforcing the effectiveness of this method in achieving high yields of carbonaceous materials.^36,37^ The lower yield obtained by pyrolysis can be attributed to the absence of sulfuric acid during the synthesis. This acid promotes the dehydration, polymerization, and carbonization processes of glycerol used as the precursor.^13^ In the absence of acid, thermal treatment led to the volatilization loss of most of the starting glycerol. Furthermore, the occurrence of redox reactions between the introduced iron and the carbon matrix may contribute to the lower yield, as the thermal treatments are known to develop porosity in materials and release carbon from the structure in the form of CO and CO_2_.^38^ Besides promoting the carbonization process, sulfuric acid modifies the surface of the solids by introducing acidic groups.^39^ Although a greater amount of acid was used in the sulfonation step following pyrolysis, there was less incorporation of acidic groups into the solids from pyrolysis compared with those from hydrothermal carbonization. This difference is evidenced by the comparative analysis of total acidity and strong acidic groups, represented by carboxylic and sulfonic groups, as shown in Table 1.

**Table 1:** Amount of Surface Acid Groups on the Carbons According to the Boehm Methodology

sample	total acid groups (mmol·g^–1^)	carboxylic + sulfonic groups (mmol·g^–1^)
HCC	4.21 ± 0.08	1.78 ± 0.10
HCC-10% Fe_3_O_4_	4.49 ± 0.04	1.99 ± 0.06
HCC-20% Fe_3_O_4_	4.89 ± 0.13	1.87 ± 0.08
CP	0.48 ± 0.06	0.12 ± 0.02
CPS	1.26 ± 0.68	0.45 ± 0.31

The number of acidic groups in the solids obtained through the hydrothermal process is similar to the results reported by Reis et al.^4^ and Mantovani et al.^40^ for carbons produced from the same precursor. The quantity of acidic groups in CP is low and decreases after the sulfonation process (CPS), indicating that the pyrolysis process generates a surface that is less prone to incorporating these sites. Previous results and literature studies on sulfonation following thermal treatment have demonstrated the need for larger amounts of sulfuric acid, and even then, the acidity results are typically lower than those obtained in this study.^41,42^ These results highlight that the *in situ* carbonization and sulfonation process promoted by sulfuric acid is a methodology that is more effective for producing modified carbons.

**Figure 2:**
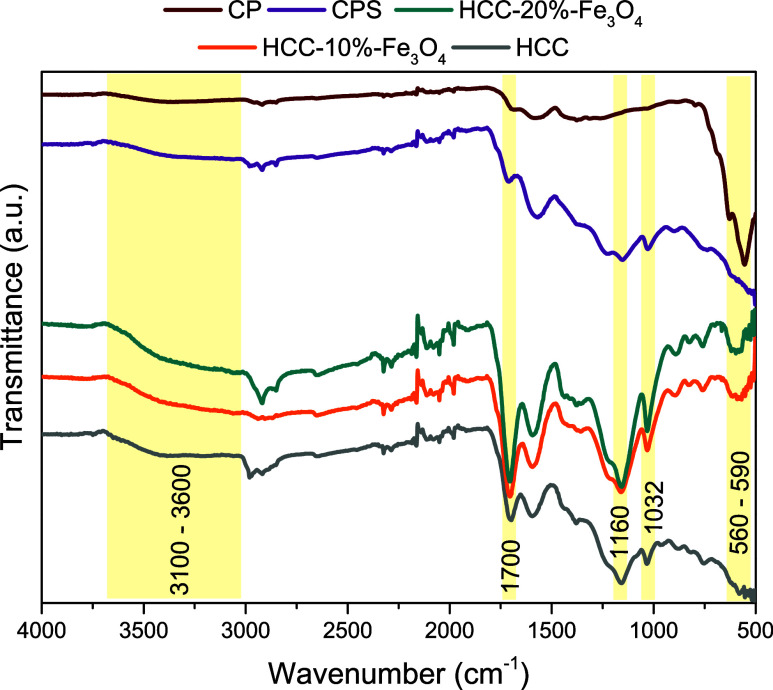
FTIR spectra of the synthesized carbons.

From the Fourier transform infrared spectroscopy (FTIR) analysis shown in Figure 2, the incorporation of oxygenated functional groups can be confirmed. Bands at 1700 cm^–1^, attributed to C=O stretching vibrations, and a broad band between 3100 and 3600 cm^–1^ corresponding to O–H vibrations, indicate the presence of carboxylic and phenolic groups.^43^ Bands at 1032 and 1160 cm^–1^ are, respectively, associated with the asymmetric and symmetric stretching of SO_2_, present in the structure of sulfonic groups.^44^ These bands are more pronounced in the solids obtained from the hydrothermal process, consistent with the Boehm titration results (Table 1).^45^ In the region of approximately 560 cm^–1^, a band of variable intensity is observed in all solids, which can be related to the Fe–O vibrations of the magnetite but also receives contributions from the carbonaceous matrix, making its attribution difficult.^34,35^

**Figure 3:**
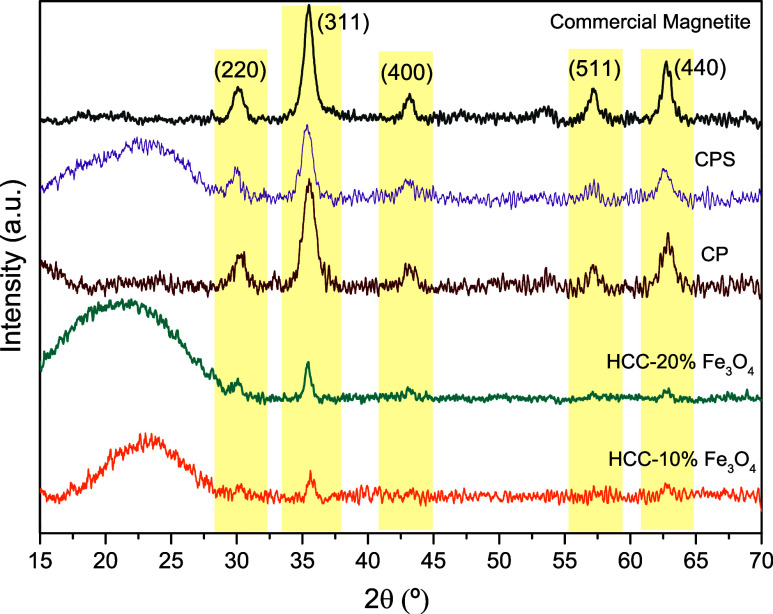
Diffractograms of synthesized solids and commercial magnetite.

This interaction between iron and oxygen in CP indicates that redox reactions occurred within the carbon matrix, capable of transforming iron nitrate into iron oxide, as described by Ndongo et al. for iron chloride.^46^ The diffractograms shown in Figure 3 demonstrate the formation of materials with crystalline regions due to the presence of iron oxide. The peaks centered at 2θ 30.2°, 35.5°, 43.2°, 57.2°, and 62.8° correspond to the (220), (311), (400), (511), and (440) planes of magnetite, a ferrimagnetic compound, which imparts magnetic properties to the solids.^32,46,47^

It is important to note that, as the lattice parameters are nearly identical, differentiating between magnetite and maghemite (γ-Fe_2_O_3_) by X-ray diffraction is quite subtle (typically, a shift of only 0.3° 2θ in the (511) Bragg peak).^48^ It is known that maghemite always accompanies magnetite in materials due to the slow oxidation of magnetite by air oxygen, resulting in a maghemite layer on its surface. Medeiros et al. identified the formation of maghemite using iron(III) nitrate as a precursor through Mössbauer spectroscopy.^32^ The reduced intensity of the bands in the diffractograms of the HCC solids demonstrates significant leaching of the magnetic phase due to the treatment with sulfuric acid. Quah et al. observed this effect with a reduction from 64% to 19.3 wt % of iron content, analyzed by energy-dispersive X-ray analysis (EDX), in a carbon derived from palm kernel shell, obtained by pyrolysis with excess iron chloride and sulfonated with sulfuric acid.^49^ Araujo et al. evaluated the reduction in the intensity of diffraction peaks corresponding to magnetite after the sulfonation process, attributing this phenomenon to the bonding of sulfonic groups.^50^ Similarly, in the case of CPS, this reduction in intensity is less pronounced compared to that of solids obtained via the hydrothermal process. This behavior can be explained by the lower insertion of Brønsted acid sites in CPS, resulting in reduced interference in the crystalline structure of magnetite and consequently a smaller decrease in the intensity of diffraction peaks.^32,49,51^

**Figure 4:**
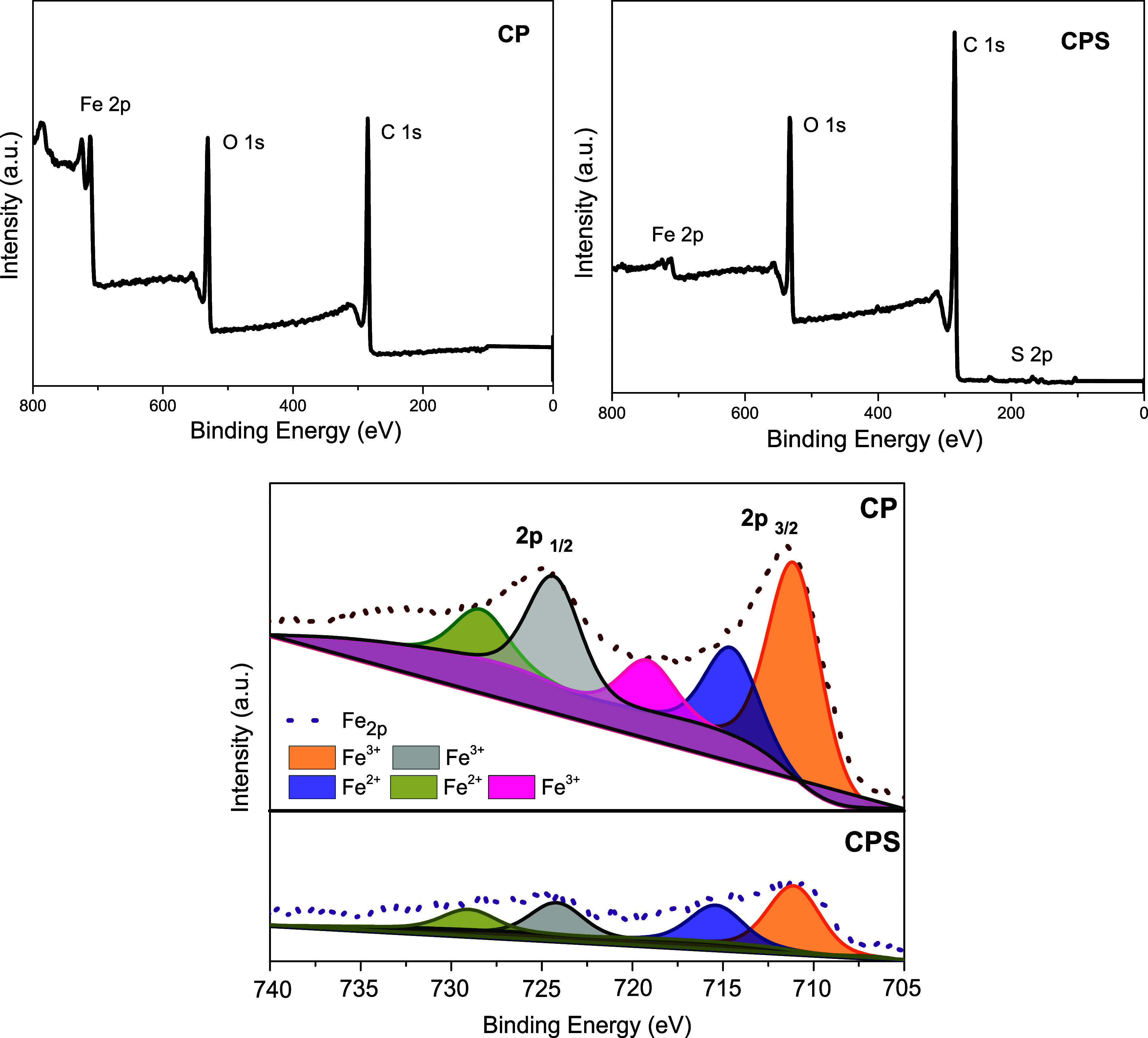
Deconvolution of the Fe_2p_ peaks of XPS spectra in CP and CPS.

The data obtained from X-ray photoelectron spectroscopy (XPS) confirm the leaching of a portion of the impregnated iron and the presence of the remaining iron. The data show iron in the oxidation states +3, with deconvolution peaks at around 711 and 724 eV, and in the +2 state, with peaks at 714 and 728 eV,^52,53^ as shown in Figure 4 for the CP and CPS solids. These data indicate the presence of magnetite, which is formed by iron ions in these two oxidation states.^51^ The deconvolution peaks of the solids obtained from the hydrothermal process in the presence of sulfuric acid are less evident (Figure S1), as the iron content in these materials is lower. Moreover, XPS analysis allowed for confirming the insertion of Brønsted acid groups, evidenced by deconvolution peaks of carbon (C_1s_) (Figure S2) centered at 284.8, 286, and 288.4 eV, which correspond to carbon–carbon double bonds, carbon bonded to hydroxyl groups, and carboxylic groups, respectively.^54^ The deconvolution of sulfur (S_2p_) (Figure S3) revealed peaks at 164.5 and 168.5 eV, related to the reduced form of sulfur (-SH) and sulfur atoms that constitute sulfonic groups.^55^

**Figure 5:**
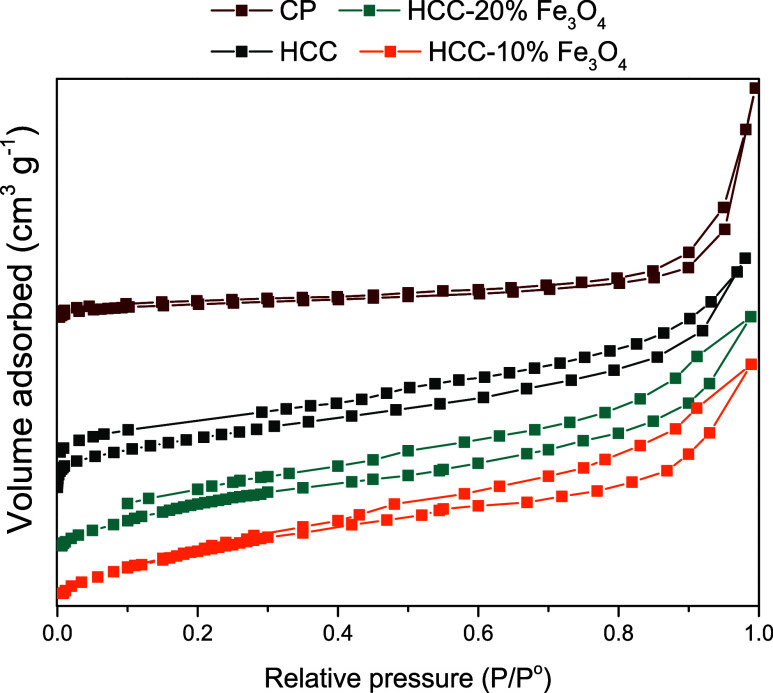
N_2_ adsorption/desorption isotherms.

It is well established that reactions capable of transforming iron salts into iron oxide occur at temperatures above 330 °C and are critical for developing the porosity and increasing the surface area. These redox reactions between iron and carbon play a crucial role in enhancing the textural properties of the material, releasing carbon from the structure as either CO or CO_2_, depending on the temperature.^38,46^ Consequently, the solids synthesized directly using commercial magnetite, subjected to lower temperatures, exhibited surface areas smaller than those obtained through pyrolysis. Higher temperatures in the pyrolysis process facilitate these redox reactions, leading to greater surface area and pore volume development.^56^ However, both surface areas are considered underdeveloped. In the case of CP, the textural properties could have been further improved if glycerol had not been vaporized during the pyrolysis process, as evidenced by the low synthesis yield. Both solids were characterized according to IUPAC classification by a combination of type I and IV isotherms, indicative of materials with the development of micropores and mesopores,^57^ as shown in Figure 5. The surface area and pore data are listed in Table 2.

**Table 2:** Textural Characteristics of the Carbons

sample	total pore volume (cm^3^·g^–1^)	micropore volume (cm^3^·g^–1^)	mesopore volume (cm^3^·g^–1^)	BET surface área (m^2^·g^–1^)
HCC	33 × 10^–3^	11 × 10^–3^	22 × 10^–3^	21
HCC-10% Fe_3_O_4_	19 × 10^–3^	53 × 10^–5^	18 × 10^–3^	13
HCC-20% Fe_3_O_4_	23 × 10^–3^	10 × 10^–6^	23 × 10^–3^	16
CP	69 × 10^–3^	19 × 10^–3^	5 × 10^–2^	39

**Figure 6:**
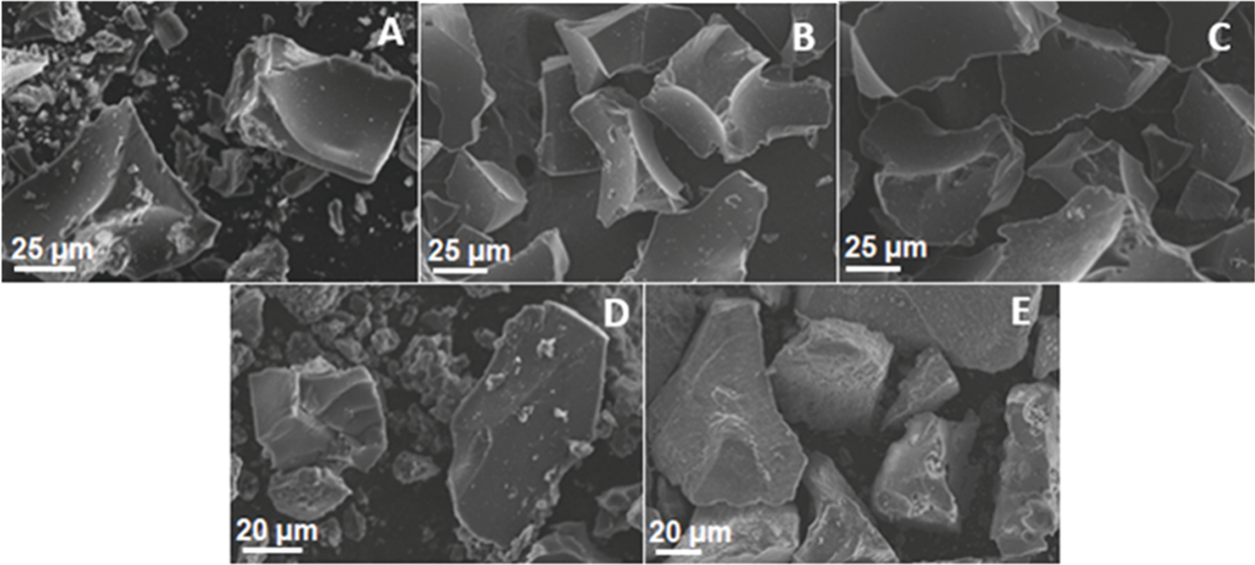
Morphology of carbons synthesized by different methodologies [(A) HCC; (B) HCC-10% Fe_3_O_4_; (C) HCC-20% Fe_3_O_4_; (D) CP; (E) CPS].

Micrographs of all of the carbons (Figure 6) did not reveal any ordering or similarity of particles, a characteristic present in amorphous materials.^58^ Elemental mapping performed through EDX analysis (Figure S4) demonstrated that carbon, oxygen, and sulfur are homogeneously distributed on the surface. However, the distribution profile of iron did not show the same behavior. These data indicate that Brønsted acid sites are uniformly anchored on the surface of the solid, and iron oxides have a nonuniform distribution, forming islands of higher concentration, especially in the samples obtained by hydrothermal carbonization.

The insertion of sulfonic groups was evaluated by three distinct techniques concerning the presence of sulfur. The highest quantification of this element was obtained by EDX, indicating a higher concentration of these groups and, consequently, of acid sites in a superficial layer (up to 2 μm).^59^ Elemental analysis, which evaluates the sulfur composition throughout the sample, and XPS, which examines superficially up to a depth of 0.01 μm,^60^ corroborate this conclusion. Table 3 presents sulfur and iron quantification evaluated by EDX and ICP-OES. This analysis confirms that iron is also present at a higher concentration in a surface layer with a significant portion leached out due to acid treatment, supporting the results previously observed by XRD and XPS.

**Table 3:** Quantification of Sulfur and Iron in the Solids

	S (%)			Fe (%)	
sample	CHNS	EDX	XPS	EDX	ICP-OES
HCC	1.35	2.50	0.39	b	b
HCC-10% Fe_3_O_4_	1.73	4.19	0.50	1.69	1.63
HCC-20% Fe_3_O_4_	1.79	5.27	0.57	3.79	3.31
CP	a	a	a	59.06	42.48
CPS	0.79	3.64	1.11	7.56	5.92

a Not detected (solid without sulfur in the composition).

b Not detected (solid without iron in the composition).

The samples obtained by pyrolysis showed higher iron contents, mainly CP, due to the large amount of iron nitrate used in the synthesis. Moreover, as previously explained, the thermal carbonization process in the absence of acid promoted substantial volatilization of glycerol, making the resulting solid more concentrated in iron. Similarly to the samples obtained by hydrothermal carbonization, the acid treatment also removed most of the iron originally present in CPS.

**Figure 7:**
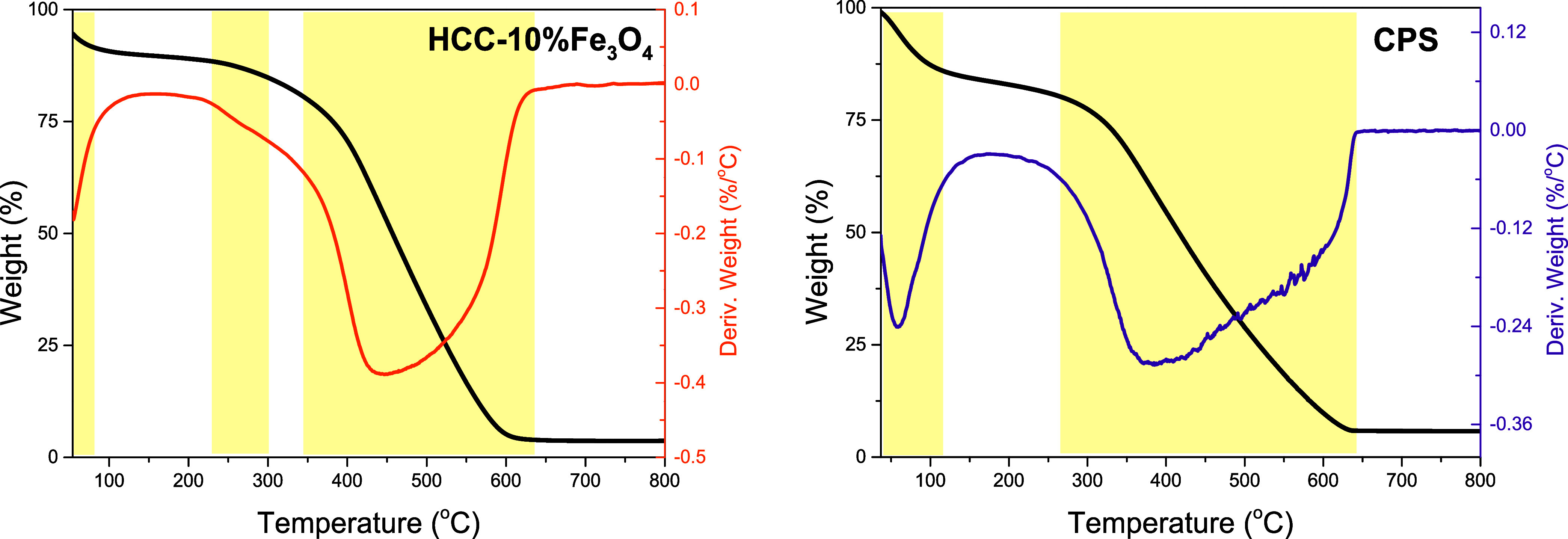
TG/DTG curves for solids obtained by the HTC and pyrolysis processes.

The thermal stability of the solids, investigated by thermogravimetric analysis (TG/DTG) in an inert atmosphere, presented in Figure 7, reveals a similar profile for both synthesis methodologies regarding the decomposition of Brønsted acid groups. The significant mass loss, evaluated at about 80% between 300 and 640 °C, is attributed to the decomposition of weaker acid groups, namely, phenolic and lactonic groups, and the decomposition of the carbonaceous matrix.^40,50,61,62^ For the HCC-10% Fe_3_O_4_ solid, a small loss of 5% between 200 and 300 °C can be observed, associated with the decomposition of stronger acid groups such as carboxylic and sulfonic groups.^63^ Both solids exhibited mass loss close to 100 °C, associated with the volatilization of gases adsorbed on the surface and primarily with moisture loss.^64^ This value corresponded to 10% for HCC-10% Fe_3_O_4_ and 15% for CPS. These data indicate that for the CPS solid, the sulfonic and carboxylic groups may have a weaker interaction with the surface and therefore decompose at lower temperatures, as the first loss region extends to approximately 140 °C. The percentage of residual mass can be associated with the impregnation of the inorganic Fe_3_O_4_ phase in the carbonaceous matrix. In the case of the HCC-10% Fe_3_O_4_ solid, the lowest quantification was observed, corresponding to about 3.69%, compared to the CP solid (Figure S5), which presented 56.6%, and the CPS, with 5.78%, following the same trend observed with the ICP-OES results. These data are consistent with the analyses obtained by XPS, ICP-OES, and XRD, which indicated lower iron impregnation using the hydrothermal carbonization methodology.

### Catalytic Tests

**Figure 8:**
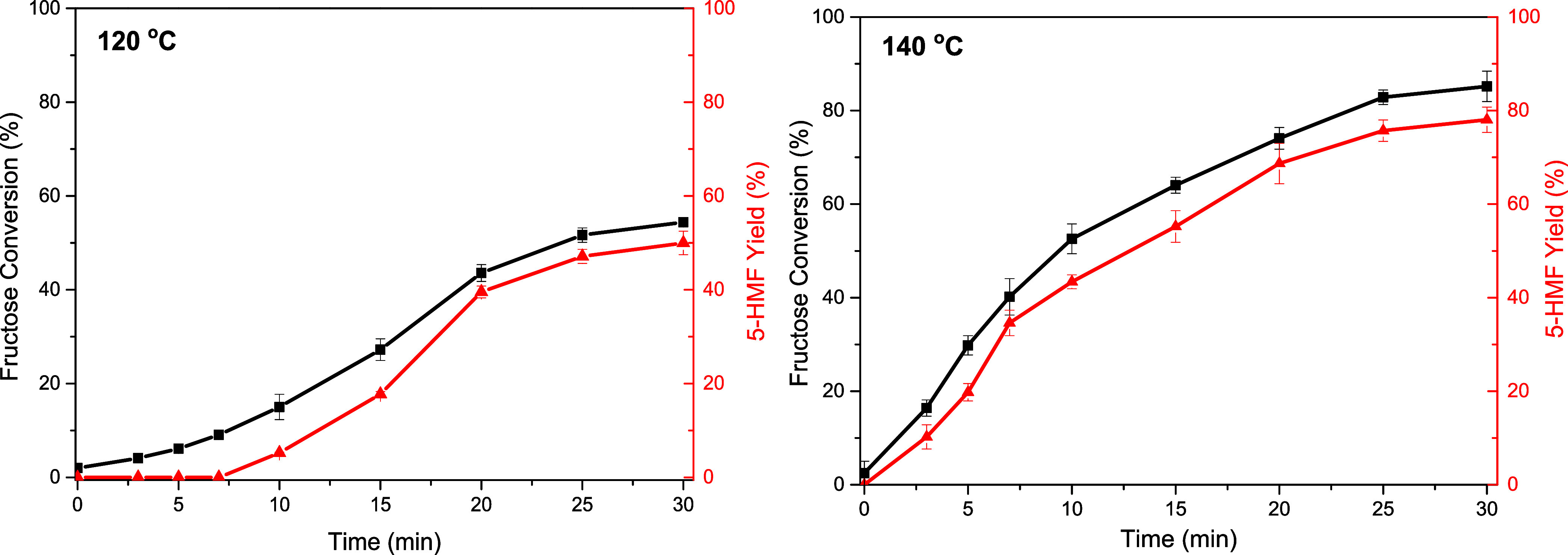
Blank test for DMSO. Conditions: 5% m/v fructose/DMSO, 120 and 140 °C.

Before the catalytic activity of the synthesized solids in the fructose dehydration reaction was evaluated, the possible activity of DMSO was checked in blank reactions, where only the substrate and the solvent were added. For this purpose, kinetic monitoring of the reaction was carried out at 120 and 140 °C. The results presented in Figure 8 demonstrate that DMSO, in addition to its primary role in facilitating reactions by dissolving reagents and stabilizing products, exhibits notable catalytic activity for the conversion of the substrate into 5-HMF.

Although the contribution of DMSO as a catalyst is ambiguously addressed in the literature, with some authors demonstrating its catalytic activity^4,6,20^ and others indicating its inertness,^21,65^ the results obtained here corroborate the hypothesis that DMSO does indeed catalyze this reaction. A plausible hypothesis was proposed by Ren et al.^18^ The authors suggested that DMSO can promote two of the three dehydration steps involved in the process. Moreover, DMSO is adept at increasing the concentration of the isomeric fructose species with the most favorable conformation for 5-HMF formation (β-d-fructofuranose form).^20^

**Figure 9:**
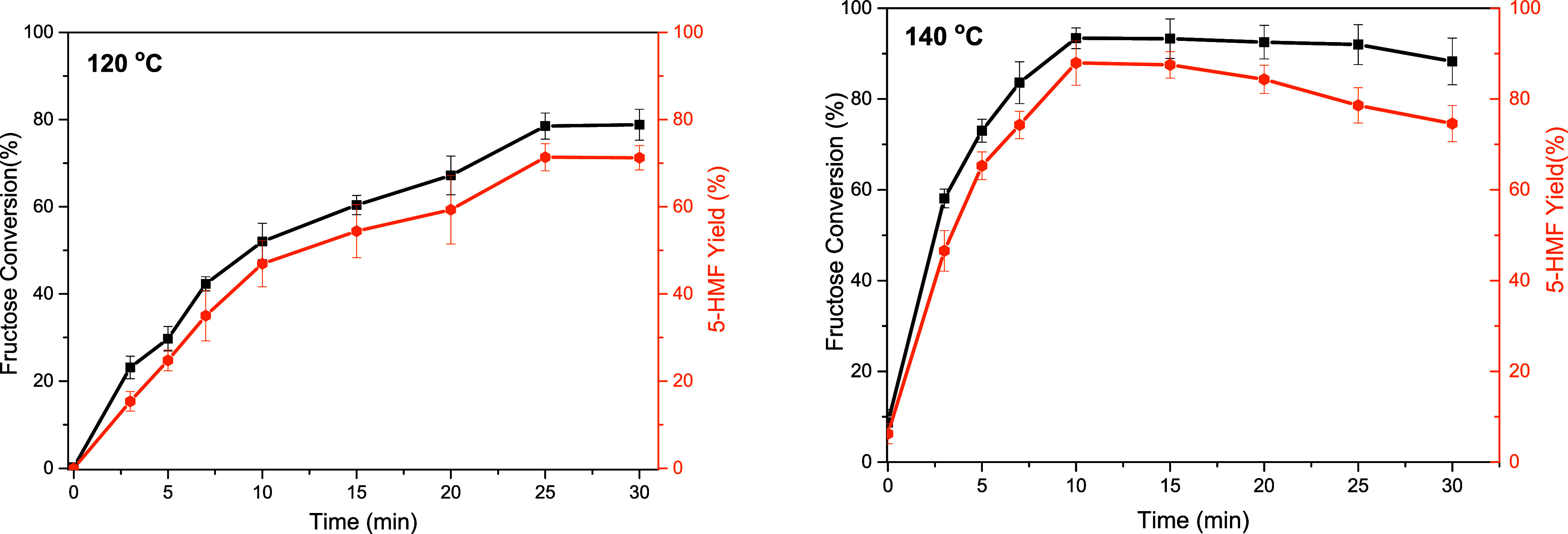
Catalytic test for HCC-10% Fe_3_O_4_. Conditions: 5% m/v fructose/DMSO, 5 wt % catalyst/substrate, 120 and 140 °C.

Even though DMSO exhibits catalytic activity under the tested conditions, especially at 140 °C, the addition of a 5 wt % catalyst containing Brønsted acidity to the reaction medium significantly increased the conversion and reaction rate. This increase can be related to the formation of catalytic species [DMSOH]^+^. When a Brønsted acid is added to the medium, the acidic hydrogen can interact with both the oxygen in DMSO and the other five oxygen atoms available in the hydroxyl groups of fructose. However, studies by Ren et al. demonstrated that the Gibbs free energy is lower when the interaction occurs with the oxygen in DMSO, forming the catalytic species [DMSOH]^+^.^18^ It is important to highlight that the temperature evaluated in this study allows for the integrity of the DMSO molecule to be maintained. Zhang et al., associated the catalytic activity with the decomposition of DMSO, in the presence of oxygen, into H_2_SO_4_.^65^ However, Whitaker et al. experimentally demonstrated that this solvent only decomposes into acidic species at temperatures above 180 °C.^20^Figure 9 presents the preliminary test with the HCC-10% Fe_3_O_4_ solid. At 120 °C, the system reached equilibrium in approximately 25 min, and compared to the blank, it increased the conversion by 27% and the yield by 24%. Raising the reaction temperature to 140 °C made this improvement even more evident, with equilibrium reached within the first 10 min. At this time, the conversion increased by 40% and the yield increased by 45% in the presence of the catalyst. Zhao et al., using the same substrate/solvent ratio as this study (5% w/v), achieved a 41% increase in conversion and a 62% increase in yield compared to the blank test under conventional heating at 140 °C for 30 min, with the addition of 20% glucose-derived carbon.^17^ Although a high yield value was achieved, the authors used a significantly higher amount of catalyst than those employed in this study (5%). Bounoukta et al. evaluated the influence of reducing the substrate/solvent ratio to 3.6% w/v. Under conventional heating at 120 °C for 120 min, they obtained a 3% increase in conversion and a 35.8% increase in yield with the addition of 11% catalyst.^6^ Given these results, this study demonstrates a greater efficacy of the catalytic system used, especially the reduced amount of catalyst and the short heating time. Superior results compared to those previously reported were achieved with just 5% catalyst and 10 min of heating.

**Figure 10:**
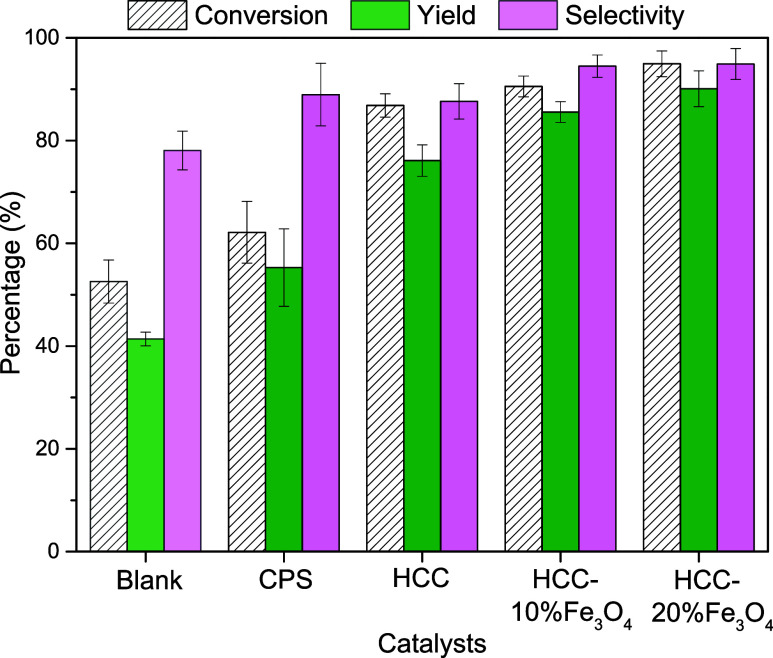
Catalytic test for solids synthesized by different methodologies, except CP. Conditions: 5% m/v fructose/DMSO, 5 wt % catalyst/substrate, 140 °C, 10 min reaction time.

Based on these results, the other solids synthesized by different methodologies were evaluated under the same optimized conditions for HCC-10% Fe_3_O_4_, corresponding to 140 °C for 10 min of reaction. The results shown in Figure 10 demonstrate that the carbons obtained through hydrothermal carbonization and in situ sulfonation showed the best performance in this study. This can be attributed to the higher number of acidic groups, as evidenced by Boehm titration, FTIR, CHNS, and EDS analyses.

Compared to the blank, CPS provided only a 10% increase in conversion and a 14% increase in yield, which are related to the low number of acidic sites, demonstrated by the low concentration of acidic sites (Table 1) and low sulfur content (Table 3).

Analyzing the turnover number (TON) and turnover frequency (TOF) values shown in Table 4, it can be observed that the HCC-20% Fe_3_O_4_ solid is the most efficient and fastest catalyst for the dehydration of fructose to 5-HMF. This efficiency can be attributed to its slightly higher content of acidic groups (Table 1) and, more importantly, to the inclusion of iron oxide, which plays a crucial role in enhancing the solid performance, especially when compared to HCC.

**Table 4:** Turnover Number (TON) and Turnover Frequency (TOF)

sample	TON (mass·mass^–1^)	TOF (mass·mass^–1^·min^–1^)
CPS	282.5	28.2
HCC	389.4	38.9
HCC-10% Fe_3_O_4_	431.8	43.2
HCC-20% Fe_3_O_4_	453.9	45.4

In addition to the TON and TOF data, which demonstrate the superiority of HCC-20% Fe_3_O_4_ compared to the other solids in this study, our material stands out compared to the literature. Although most studies investigating acidic carbons in the fructose dehydration process use conventional heating, some research deserves attention for using microwave heating. Wang et al. achieved 91% conversion and 79% yield after 5 min at 150 °C, using twice the amount of catalyst and 72% more H_2_SO_4_ during the synthesis process compared to our study.^54^ In another study, 88% conversion and 71% yield were achieved using acidic catalysts containing both W and Mo, supported on zirconia. A 10% by weight of these catalysts was used under reaction conditions of 5 min at 140 °C.^21^ Doan et al. employed acid-functionalized carbonaceous catalysts and achieved a 79.9% yield of 5-HMF in 20 min. Unlike the previous studies, which used a 5% substrate/solvent ratio (w/v), they utilized a ratio of 18%. Additionally, the amount of catalyst used was 0.6% by weight, and the temperature for microwave heating was not explicitly stated, as the authors reported results based on power settings.^22^ Given these results, the HCC-20% Fe_3_O_4_ solid stands out by converting 94% of the fructose in 90% yield and 95% selectivity in just 10 min, using a lower amount of catalyst and milder conditions.

The combined use of acidic sites, iron oxide, and microwaves (MW) is key to understanding the catalytic efficiency achieved with HCC-20% Fe_3_O_4_. Unlike conventional heating, which relies on conduction and convection to first raise the temperature of the container walls and the outer layers, microwave heating allows for direct interaction with the reaction medium, specifically with the catalyst, through intermolecular collisions and relaxation.^66^ This interaction occurs due to the presence of irradiation absorbers, which can convert electromagnetic energy into heat. This process is driven by the dielectric properties of the materials, including their ability to absorb energy (dielectric constant) and their potential to dissipate it as heat (dielectric loss).^23^ The presence of these absorbers leads to the formation of hot spots, regions where the temperature is significantly higher than the surrounding environment,^67,68^ a phenomenon that has proven effective in 5-HMF production. In general, carbon-based materials are good MW absorbers, but surface modifications are necessary to enhance this effect. Kong et al. and Ji et al. experimentally demonstrated that the introduction of sulfonic groups in carbonaceous materials promotes the formation of hot spots through synergistic interaction with microwaves, improving energy efficiency and increasing the yield of 5-HMF.^26,27^ This selective surface heating was also evaluated by Lyu et al., who showed that the polarization promoted by acidic sites (sulfonic and carboxylic) can rapidly rotate in the microwave field, increasing the likelihood of collisions between substrate molecules and the catalyst, thereby improving catalysis.^28^ In our recent investigation,^4^ we observed that the production of 5-HMF using acidic carbons is significantly enhanced when microwaves are employed as the heating method, compared to conventional techniques. Although not previously discussed, the acidic groups played a dual role by acting both as catalytic sites and as microwave (MW) absorbers, contributing to the improved efficiency observed in the results.

The introduction of iron as an oxidized species also can polarize material surfaces. Iron oxide exhibits distinct characteristics of reflection and polarization. This means that when exposed to electromagnetic fields, such as microwaves, its particles respond in different ways such as the alignment of internal dipoles and wave reflection. These responses generate multiple relaxations that are various modes of electromagnetic energy dissipation within the material. These processes enhance the material’s ability to absorb microwaves, allowing for more efficient energy conversion.^29,69^ Zhang et al. demonstrated that nanofibers composed of Fe_3_O_4_/C exhibit improved microwave absorption properties compared to pure carbon nanofibers due to the introduction of Fe_3_O_4_ nanoparticles.^70^ Similarly, Liu et al. showed that Fe_3_O_4_ displays dielectric behaviors, responding to microwaves in the frequency range of 2–18 GHz.^30^ Meng et al. evaluated the dielectric behaviors of Fe_3_O_4_ nanoparticles coated with an ultrathin carbon layer and concluded that large interface areas can significantly enhance dielectric loss capacity, playing a crucial role in microwave absorption performance.^71^ This indicates that the more exposed the particles are to microwaves, the greater may be the absorption efficiency may be. This factor is particularly relevant in our study, as EDX analysis revealed higher iron concentrations in surface layers up to 2 μm deep (Table 3). To understand the effect of iron in our catalysts, we evaluated the conversion of fructose under conventional heating at 100 °C for 1 h using the same quantities of substrate and catalyst as in the microwave tests. The HCC-20% Fe_3_O_4_ solid was able to convert approximately 46% of the fructose, with a yield and selectivity of 34 and 74%, respectively, for 5-HMF. Compared to Figure 10, these data demonstrate that both the acidic sites and the iron can absorb microwave radiation, thereby increasing reaction efficiency.

Additionally, compared to our previous study,^4^ we observed an increase in selectivity in both heating methods, which can be attributed to a combination of factors. First, the solids obtained through the hydrothermal method did not undergo an activation process and, therefore, did not develop a microporous structure (Table 2). This prevented intraparticle transport effects, which difficult both the entry of reagents into the active acidic sites within the pores and the exit of already formed products from the pores, facilitating their polymerization into insoluble humins. Microwave heating is more efficient than conventional heating, significantly reducing reaction time. Thus, the contact time between species in the reaction medium was also reduced, limiting the formation of humin.

Additionally, the possible influence of Lewis acid sites on the solids should be highlighted. In a recent study, intermediate species were identified in the formation of highly condensed humin structures.^72^ These are compounds with a low level of cross-linking, formed by furan rings interconnected by short aliphatic chains containing a wide range of functionalities, including alcohols, ethers, carboxylic acids, esters, aldehydes, and ketones.^73,74^ Like the process generally accepted for glucose isomerization,^75^ the presence of Lewis acidity can substantially limit the formation of humins. Lewis acid centers can withdraw electron density from carbonyl groups, present in both glucose structures and humin precursors, making their carbon more electrophilic and therefore more susceptible to attack by a hydride species (Lewis acid-catalyzed intramolecular hydrogen shift) and subsequent dehydration catalyzed by Brønsted acids.^76,77^ Indeed, reduced metal oxides generate oxygen vacancies, which act as Lewis acid sites to activate oxygen atoms in lignin and facilitate the selective cleavage of CO bonds through hydrogenolysis and hydrodeoxygenation reactions.^78,79^ To briefly understand these oxygen vacancies, we conducted a preliminary test under conventional heating at 150 °C for 60 min and found that HCC-20% Fe_3_O_4_ can isomerize approximately 76% of the glucose used as a substrate.

Thus, the dehydration process promoted by Brønsted sites is facilitated, preventing the formation of highly cross-linked and insoluble humin structures. It is worth noting that in situations where only Lewis acid sites are present, fructose can decompose and also produce humins due to autopolymerization or cross-condensation, meaning the beneficial effect of Lewis sites only occurs in the presence of Brønsted sites.^80,81^

To investigate whether iron, as a Lewis acid site, could exhibit any activity, the CP solid was evaluated under the optimized conditions. This solid, composed mainly of carbon and iron and without sulfonic group insertion, inhibited the fructose dehydration reaction. The conversion was below 15%, and the amount of 5-HMF produced was below the HPLC detection limit. Thus, although the amount of fructose recovered at the end of the reaction was lower than the initial amount, no detectable product was formed. This outcome is likely due to the adsorption of the substrate within the larger pore volume of this solid, particularly in the mesopores, compared with other materials with less developed surface areas (Table 2). The enhanced adsorption reduced the concentration of fructose in the reaction medium, suggesting that it was not converted. Additionally, the magnetic properties of CP, which was not acid-treated and thus had a high iron content as assessed by ICP-OES, may have also influenced the process. The strong electromagnetic interaction between the CP and the magnetic stir bar could have caused the CP to be strongly retained on its surface, further limiting contact with the substrate. These results reinforce the idea that Brønsted acid sites are crucial for promoting fructose dehydration.

### Catalyst Recycling

**Figure 11:**
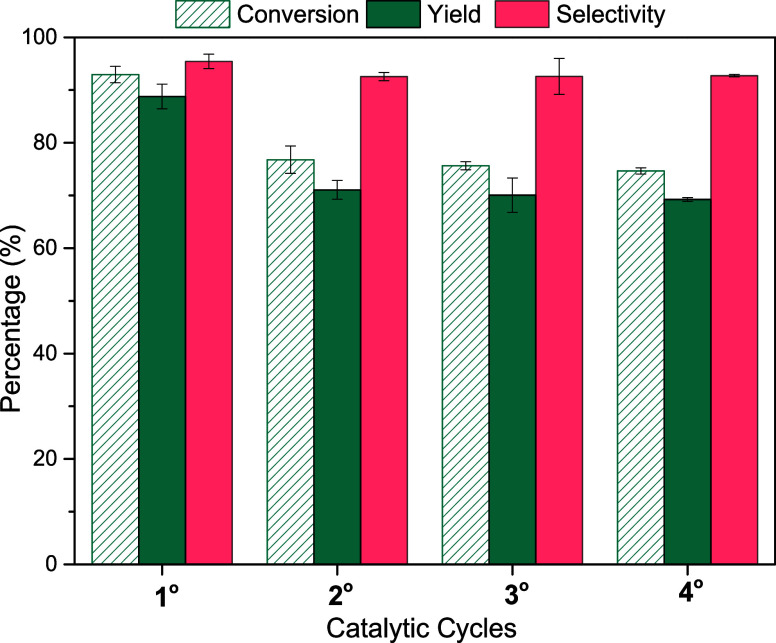
Recycling test for HCC-20% Fe_3_O_4_. Conditions: 5% m/v fructose/DMSO, 5 wt % catalyst/substrate, 140 °C, 10 min reaction time.

The feasibility of reuse and the stability of the HCC-20% Fe_3_O_4_ catalyst were investigated through consecutive catalytic cycles under the optimized reaction conditions. The results presented in Figure 11 demonstrate a decline in catalytic activity only after the first cycle with a decrease of approximately 16% in conversion and 2% in selectivity. From the second cycle onward, the activities remained stable for two consecutive cycles, maintaining a high selectivity of around 93%. These results suggest stabilization of the catalyst and consistency in efficiency over subsequent cycles.

The observed decrease after the first use has been reported in our previous studies^4,7,82^ and the literature on other carbonaceous materials.^83−85^ It is generally associated with the leaching of acidic groups and the deposition of humins or other organic compounds.

For HCC-20% Fe_3_O_4_, the formation of organic compounds such as formic acid or levulinic acid was not detected, and selectivity remained high from the first use, indicating the significant absence of humins. The reduction after the first cycle may be related to the loss of residual sulfuric acid retained in the material’s pores and not completely removed during washing in the synthesis step. The activity maintenance after the second cycle suggests that the catalyst stabilized, with the remaining acidic groups stable and integrated into the solid matrix. FTIR analyses (Figure S6), CHNS (Table S1), and EDX (Figure S7) corroborate these observations, showing the preservation of sulfonic and carboxylic groups and the homogeneous distribution of sulfur on the surface throughout the cycles, supporting the efficacy and consistency of the acidic group insertion process.

### Leaching Test

**Figure 12:**
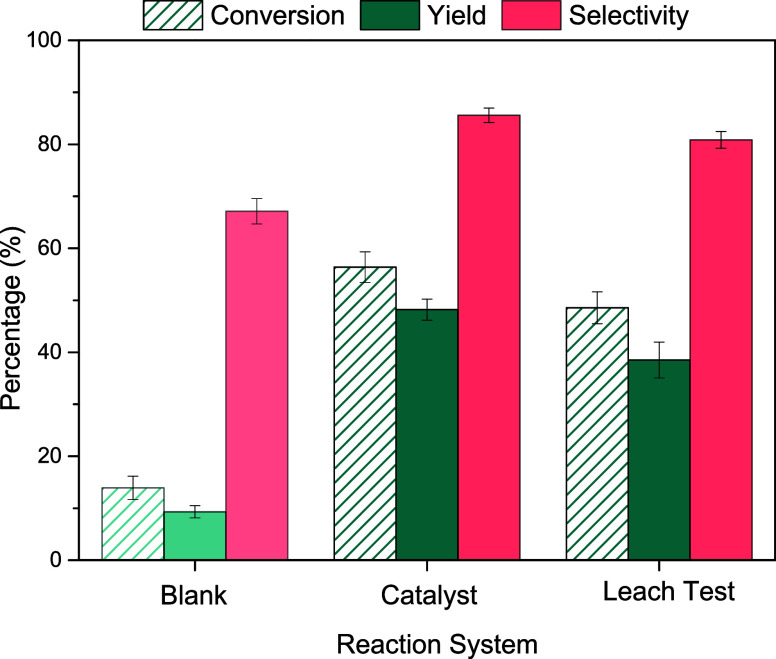
Leaching test for HCC-20% Fe_3_O_4_. Conditions: 5% m/v fructose/DMSO, 5 wt % catalyst/substrate, 140 °C, 3 min reaction time.

A leaching test was conducted to verify the presence of residual sulfuric acid in the HCC-20% Fe_3_O_4_ catalyst, with the results shown in Figure 12.

The results indicate that part of the acidic species responsible for catalysis was transferred from the carbon to the liquid phase. After the solid removal, the fructose dehydration reaction continued, achieving results slightly lower than those obtained with the catalyst. The sulfuric acid may have been retained in the solid’s structure during synthesis and was subsequently released into the solvent during the first heating process. The solvent containing the residual acid promoted catalysis, even after removing the catalyst from the medium. The sustained heterogeneous catalytic activity from the second cycle onward, driven solely by the acidic sites firmly anchored to the solid and observed with the use of a new aliquot of solvent in each cycle, leads to the conclusion that all free acid was transferred to the liquid phase after the initial heating of the solid with DMSO.

A second leaching test was conducted using the catalyst recovered by filtration after the initial test to ensure no further leaching of acidic species after the first reaction. The recovered catalyst was dried at 100 °C and subjected to another catalytic activity test in the absence of the substrate. In this second leaching test, the amount of 5-HMF detected was the same as in the blank test, confirming the absence of additional catalytic activity due to the [DMSOH]^+^ species formed from the free acids. This confirms that the free acid originally present in the solid was completely removed during the first leaching test.

The dehydration mechanism can be associated with both the presence of these free acids and the acidic sites embedded in the surface of the matrix. According to the theoretical studies by Ren et al., dehydration can occur through the action of DMSO as well as the involvement of the catalytic species DMSOH^+^, with the catalytic route via DMSOH^+^ considered thermodynamically more stable than the route via DMSO.^18^ The formation of DMSOH^+^ can occur through the interaction of the solvent with the acidic groups of the catalyst via hydrogen bonding or through the free acids that were initially adsorbed in the pores of the solid. The interaction of these catalytic species with the substrate promotes the formation of cyclic structures, facilitating the removal of a water molecule and enabling regeneration of the catalyst for new reaction cycles.

## Conclusions

Chemically modified carbons were produced by using pyrolysis and hydrothermal carbonization methods. Solids obtained via the hydrothermal process outperformed those from pyrolysis due to higher synthesis yield, milder conditions of preparation, and greater incorporation of Brønsted acid sites with reduced use of sulfuric acid. Although significant leaching of the magnetic phase occurred when sulfuric acid was used, this effect was more pronounced in samples with higher concentrations of Brønsted acid sites. Nevertheless, both solids produced through hydrothermal carbonization contained iron impregnated in the oxide form. The combination of Lewis and Brønsted acid sites and the heating method was crucial in selectively promoting fructose dehydration to 5-HMF, achieving 90% yield with approximately 95% selectivity in just 10 min at 140 °C. Recycle and leaching tests demonstrated catalyst stability postremoval of residual free acid retained in material pores during synthesis. The study provides valuable insights for advancing effective catalyst production in the fructose dehydration process.

## Supporting Information Available

Characterization analyses (XPS, N_2_ adsorption, MEV-EDS, TG/DTG) (PDF)

## Abbreviations

5-HMF: 5-hydroxymethylfurfural
DMSO: dimethyl sulfoxide
HTC: hydrothermal carbonization
FTIR: Fourier transform infrared spectroscopy
ATR: attenuated total reflectance
ICP-OES: Inductively coupled plasma optical emission spectroscopy
XPS: X-ray photoelectron spectroscopy
TG: thermogravimetric analysis
DTA: differential thermogravimetric analysis
HPLC: high-performance liquid chromatography
RID: refractive index detector
DAD: diode array detector
EDX: energy-dispersive X-ray analysis
XRD: X-ray diffraction
CHNS: elemental analysis
