# Supplementary material for: Iron-Modified Acid Carbons for the Conversion of Fructose to 5-Hydroxymethylfurfural under Microwave Heating
Source: ACS Omega. 2024 Nov 1;9(45):45328–41. doi: 10.1021/acsomega.4c07030 (PMC11561637; doi:10.1021/acsomega.4c07030)
Supplement: Supplementary file 1 — ao4c07030_si_001.pdf [file ao4c07030_si_001.pdf]

Iron-modified acid carbons for the conversion of fructose to 5-hydroxymethylfurfural under microwave heating

Letícia F. L. Machado[1]; Luana S. Andrade[1,2]; Dalmo Mandelli[1]; Wagner A. Carvalho[1]*

[1] Center for Natural Sciences and Humanities, Federal University of ABC (UFABC), Av. Dos Estados, 5001, Santo André – SP, CEP 09210580, Brazil

[2] Department of Chemistry Northwestern University, 2145 Sheridan Road, Evanston, Illinois 60208, USA

[*] Corresponding author: Phone/fax: +55 (11) 4996-8386
E-mail address: wagner.carvalho@ufabc.edu.br

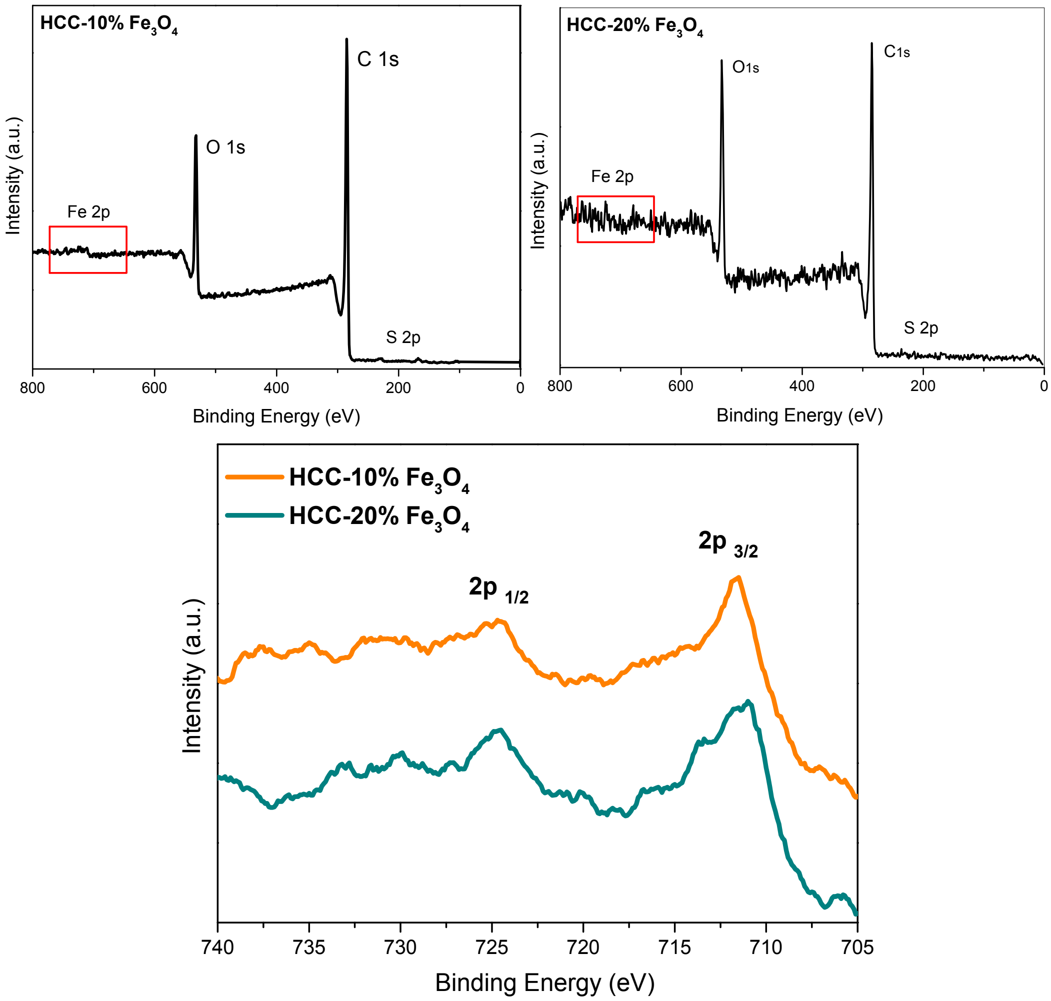

Figure S1: XPS Spectrum and deconvolution of the Fe_{2p} peaks spectra in HCC-10% Fe_3O_4 and HCC-20% Fe_3O_4

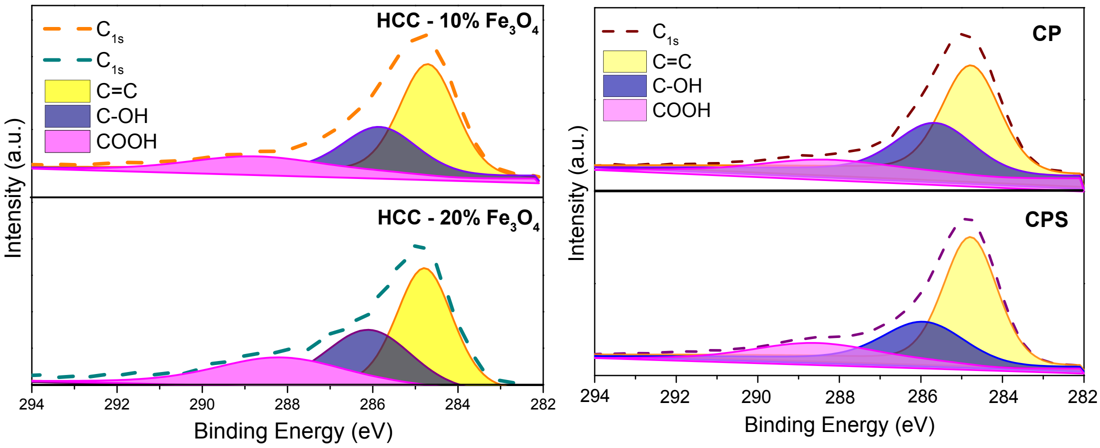

Figure S2: Deconvolution of the C_{1S} peaks spectra in HCC-10%Fe_3O_4, HCC-20%Fe_3O_4, CP and CPS

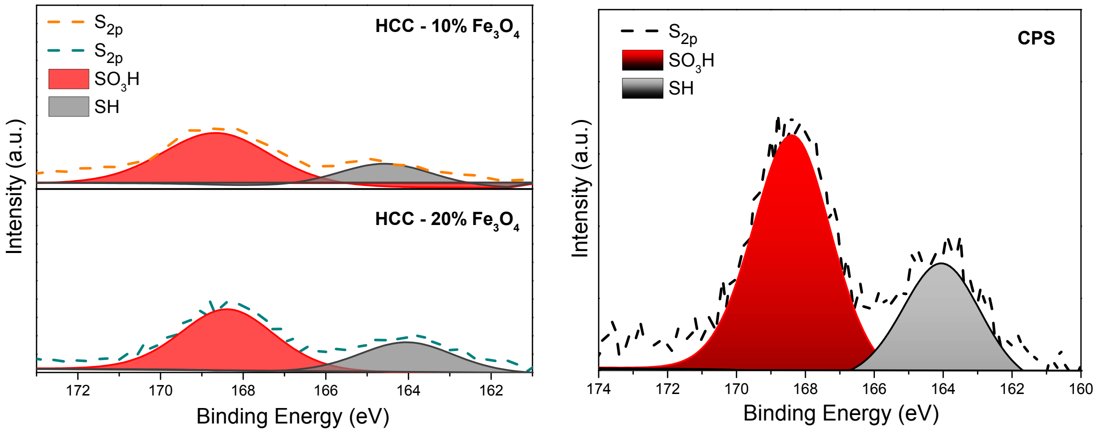

Figure S3: Deconvolution of the S_{2p} peaks spectra in HCC-10%Fe_3O_4, HCC-20%Fe_3O_4, CP and CPS

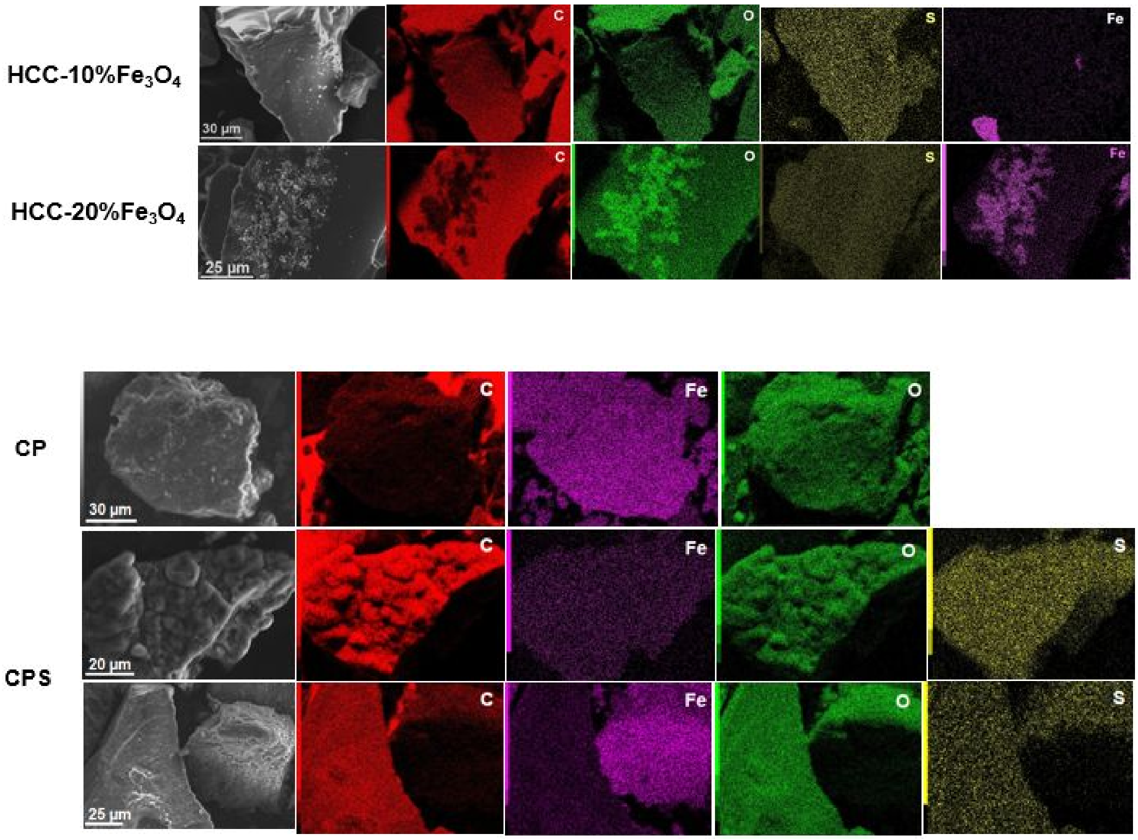

Figure S4: Morphology of coals synthesized by different methodologies

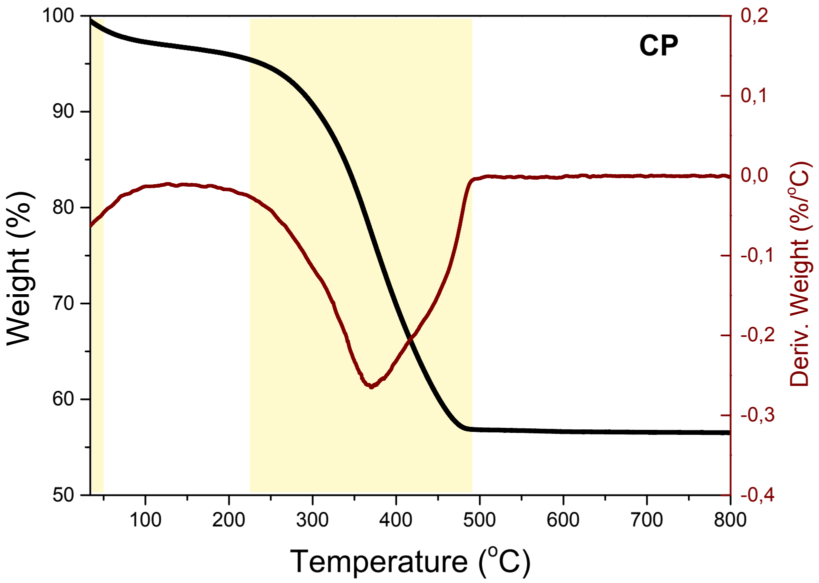

Figure S5: TGA/DTA curve for CP

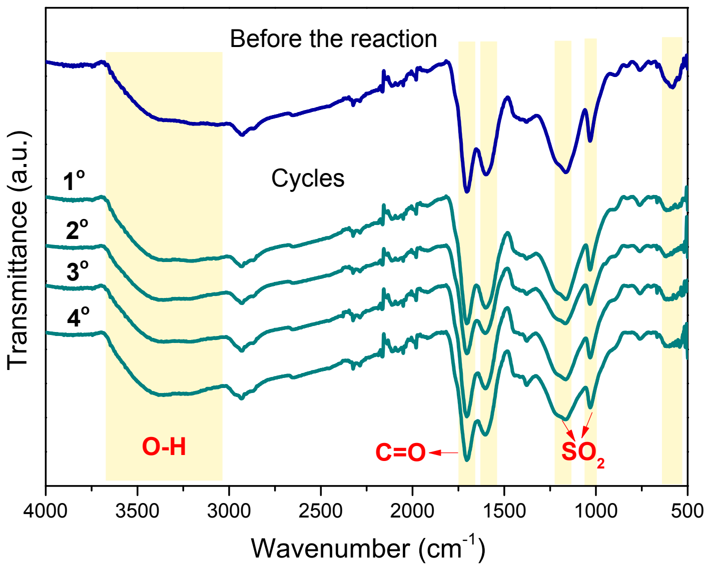

Figure S6: FTIR spectra of HCC-20%Fe_3O_4 before and after use

Table S1: Elemental sulfur composition before use and after 4 consecutive cycles

Sample HCC-20% Fe_3O_4	Weight (mg)	%S
Before use	2,34 ± *0,02*	**1,79 ± *0,04***
1º Cycle	2,43 ± *0,03*	**0,95 ± *0,02***
2º Cycle	2,32 ± *0,05*	0,90 ± *0,05*
3º Cycle	2,41 ± *0,02*	0,88 ± *0,07*
4º Cycle	2,48 ± *0,04*	0,87 ± *0,11*

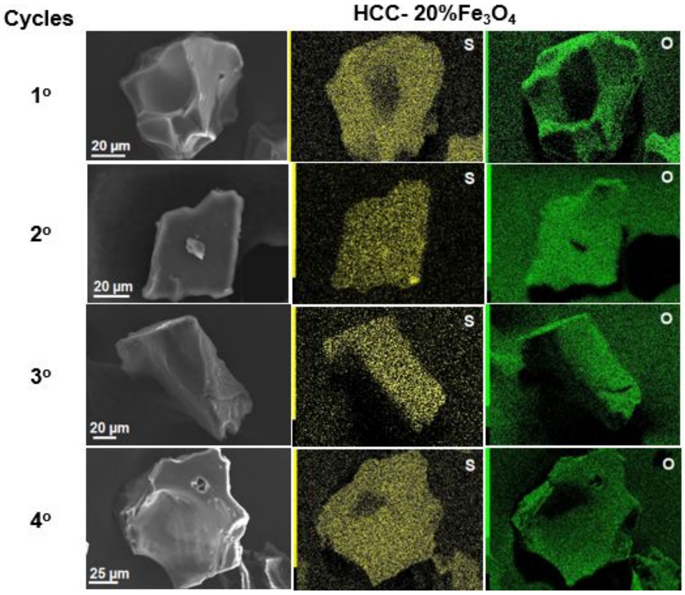

Figure S7: Dispersion analysis (EDX) of oxygen and sulfur elements after each reaction cycle
